# Angiography-Based 4-Dimensional Superficial Wall Strain and Stress: A New Diagnostic Tool in the Catheterization Laboratory

**DOI:** 10.3389/fcvm.2021.667310

**Published:** 2021-06-18

**Authors:** Xinlei Wu, Masafumi Ono, Hideyuki Kawashima, Eric K.W. Poon, Ryo Torii, Atif Shahzad, Chao Gao, Rutao Wang, Peter Barlis, Clemens von Birgelen, Johan H.C. Reiber, Christos V. Bourantas, Shengxian Tu, William Wijns, Patrick W. Serruys, Yoshinobu Onuma

**Affiliations:** ^1^Institute of Cardiovascular Development and Translational Medicine, The Second Affiliated Hospital of Wenzhou Medical University, Wenzhou, China; ^2^Department of Cardiology, National University of Ireland Galway (NUIG), Galway, Ireland; ^3^Smart Sensors Lab, National University of Ireland Galway (NUIG), Galway, Ireland; ^4^Department of Cardiology, Academic Medical Center, University of Amsterdam, Amsterdam, Netherlands; ^5^Department of Medicine, Melbourne Medical School, St Vincent's Hospital, University of Melbourne, Melbourne, VIC, Australia; ^6^Department of Mechanical Engineering, University College London, London, United Kingdom; ^7^Department of Cardiology, Xijing Hospital, Xi'an, China; ^8^Faculty of Medicine, Dentistry Health Sciences, Melbourne Medical School, University of Melbourne, Melbourne, VIC, Australia; ^9^Thoraxcentrum Twente, Medisch Spectrum Twente, Enschede, Netherlands; ^10^Department of Health Technology and Services Research, Technical Medical Centre, Faculty of Behavioural, Management, and Social Sciences, University of Twente, Enschede, Netherlands; ^11^Department of Radiology, Leiden University Medical Center, Leiden, Netherlands; ^12^Institute of Cardiovascular Science, University College London, London, United Kingdom; ^13^Department of Cardiology, Barts Heart Centre, London, United Kingdom; ^14^School of Biomedical Engineering, Biomedical Instrument Institute, Shanghai Jiao Tong University, Shanghai, China; ^15^Imperial College London, National Heart and Lung Institute, London, United Kingdom

**Keywords:** invasive coronary angiography, coronary artery dynamics, superficial wall strain, quantitative assessment method, computational coronary pathophysiology

## Abstract

A novel method for four-dimensional superficial wall strain and stress (4D-SWS) is derived from the arterial motion as pictured by invasive coronary angiography. Compared with the conventional finite element analysis of cardiovascular biomechanics using the estimated pulsatile pressure, the 4D-SWS approach can calculate the dynamic mechanical state of the superficial wall *in vivo*, which could be directly linked with plaque rupture or stent fracture. The validation of this approach using *in silico* models showed that the distribution and maximum values of superficial wall stress were similar to those calculated by conventional finite element analysis. The *in vivo* deformation was validated on 16 coronary arteries, from the comparison of centerlines predicted by the 4D-SWS approach against the actual centerlines reconstructed from angiograms at a randomly selected time-point, which demonstrated a good agreement of the centerline morphology between both approaches (scaling: 0.995 ± 0.018 and dissimilarity: 0.007 ± 0.014). The *in silico* vessel models with softer plaque and larger plaque burden presented more variation in mean lumen diameter and resulted in higher superficial wall stress. In more than half of the patients (*n* = 16), the maximum superficial wall stress was found at the proximal lesion shoulder. Additionally, in three patients who later suffered from acute coronary syndrome, the culprit plaque rupture sites co-localized with the site of highest superficial wall stress on their baseline angiography. These representative cases suggest that angiography-based superficial wall dynamics have the potential to identify coronary segments at high-risk of plaque rupture and fracture sites of implanted stents. Ongoing studies are focusing on identifying weak spots in coronary bypass grafts, and on exploring the biomechanical mechanisms of coronary arterial remodeling and aneurysm formation. Future developments involve integration of fast computational techniques to allow online availability of superficial wall strain and stress in the catheterization laboratory.

## Introduction

**Figure 1:**
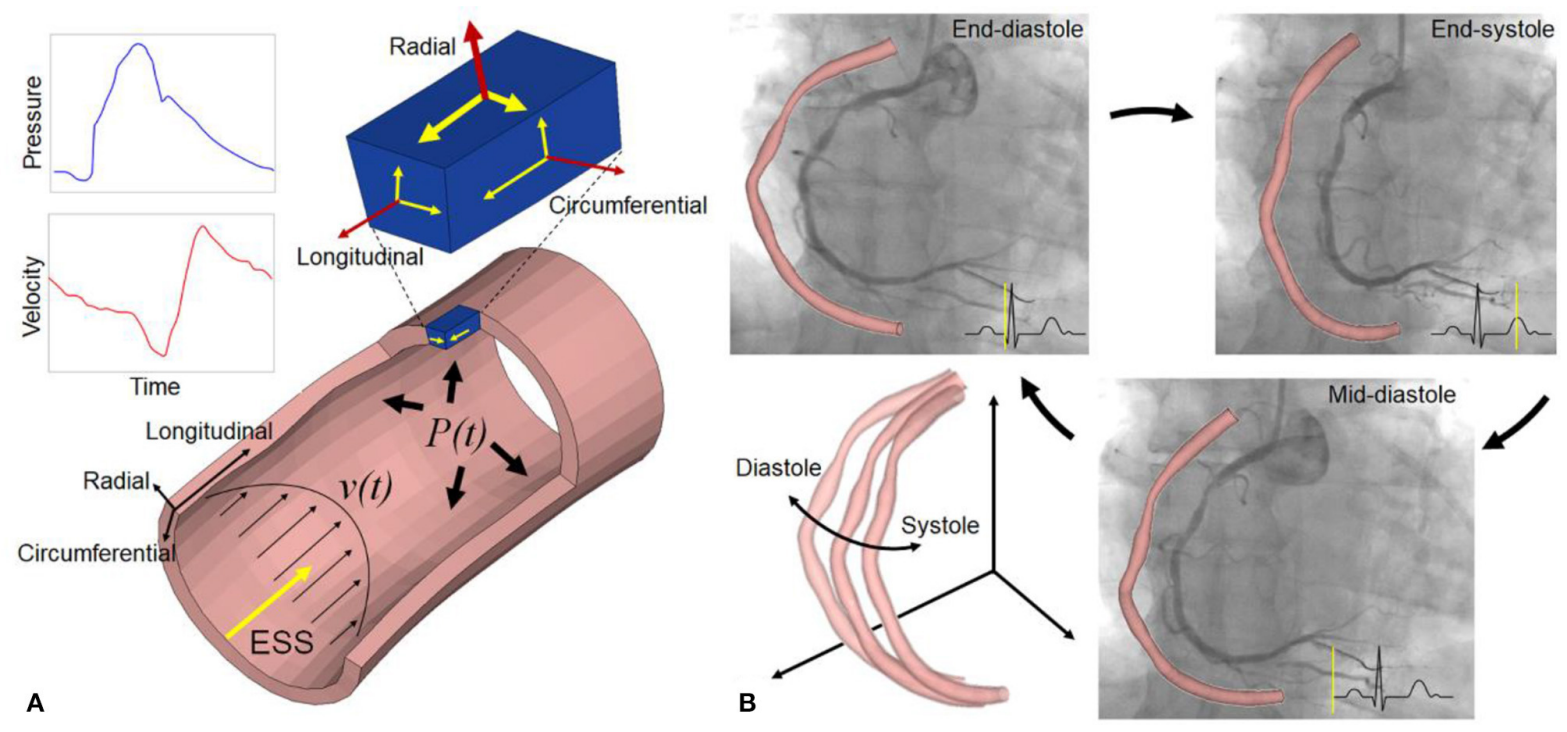
Biomechanical forces and dynamic coronary arterial motion. **(A)** Time-varying blood flow induces endothelial shear stress (ESS) along the vessel lumen. Radial (normal) strain/stress caused by intraluminal blood pressure is perpendicular to the endoluminal wall (top surface of a small material volume, blue color), and two shear strain/stress couples also exist in circumferential and longitudinal directions on this surface. The volume has two other orthotropic surfaces with strain/stress (small arrows), such that a total of nine components are operative. Red and yellow arrows refer to normal and shear strain/stress, respectively. As an equivalent quantity without direction, von Mises stress and strain, calculated from the nine components, is used to describe the complex multi-dimensional mechanical state for easy understanding and application in clinical fields (1). **(B)** Coronary angiography and 3D geometries at three specific cardiac phases: end-diastole, end-systole, and mid-diastole. It shows the large cyclic motion and deformation of a right coronary artery in 3D space.

Coronary arteries are continuously subjected to biomechanical forces, including myocardial contraction and relaxation, intraluminal pulsatile blood pressure, flow drag forces, and constrained by surrounding tissues. These biomechanical forces generate dynamic strain and stress on the coronary arterial wall (Figure 1A). The dynamic wall stress induced by cyclic deformation, which is around 10^3^~10^5^ times greater than fluid-induced endothelial shear stress (ESS) (2), can trigger the rupture of atherosclerotic fibrous cap and disruption of inflamed vulnerable plaque (3). With increased severity and extent of coronary artery disease, the vascular dynamic deformation performance could be deteriorated due to the loss of elasticity (4). Furthermore, the dynamic stress and deformation of atherosclerotic coronary arterial walls (Figure 1B) are particularly relevant to vascular remodeling acute clinical events, such as myocardial infarction or unstable angina (3), as well as the biomechanical compatibility of implanted stents and scaffolds (5).

The deformation of coronary arteries *in vivo* can be quantitatively measured by mechanical strain. The mechanical strain of arterial wall is defined as the stretching or compressing, and angular deformation relative to its predefined reference state. This wall strain caused by vessel deformation can be assessed from modern standard X-ray coronary angiography. Taking into account the maximum speed of 34.5–250.0 mm/s of the arterial motion in early systole (6, 7), coronary angiography with high frame rate (15–30 frames/s) and high spatial resolution (150–250 μm) can indeed capture the deformation of the vascular wall (8, 9). Only two angiographic image runs with different projections are needed to perform computation of four-dimensional superficial wall strain (4D-SWS) of coronary arteries (10, 11), since the dynamic deformation results from the various biomechanical forces, including cardiac contraction and blood pressure. The periodic variation in coronary arterial diameter ~10–15% caused by pressure pulsation (12), which can be captured by angiography. Note that SWS is derived as a combination of radial diameter variation as well as deformation in the longitudinal and circumferential directions. Assuming specific material properties for the normal and stenotic arterial segments, dynamic superficial wall stress can be further calculated.

An attractive feature of this approach is an inverse computation method that the cyclic motion of coronary arteries *in vivo* is used to calculate the dynamic strain and stress of arterial walls for intraprocedural on-line computation and analysis. The arterial motion and deformation represent the resultant of various complex biomechanical and physiological alterations, including pulsatile blood pressure, vessel stretching, bending and twisting, and acting on both normal and diseased vessel segments, each with different wall composition and mechanical properties. Another promising feature of this approach is that it focuses on the biomechanical state of the superficial layer of vessel wall (i.e., the interface between lumen and subendothelial layer), which could be directly linked with plaque rupture or stent fracture.

To further understand the biomechanical triggering mechanisms of acute coronary events and eventually improve the prediction of future events, it may be of paramount importance to take SWS into account. Indeed, the angiography-based SWS reflects the dynamic deformation of coronary arteries during the cardiac cycle and “hot spots” may identify coronary segments at higher risk of plaque rupture or dissection. This review highlights the concept and validation of this new method and its potential value in identifying vulnerable coronary plaque and therefore at high risk of acute disruption or rapid disease progression.

## Calculation Mehtods of Angiography-Based 4D Coronary Artery Dynamics

**Figure 2:**
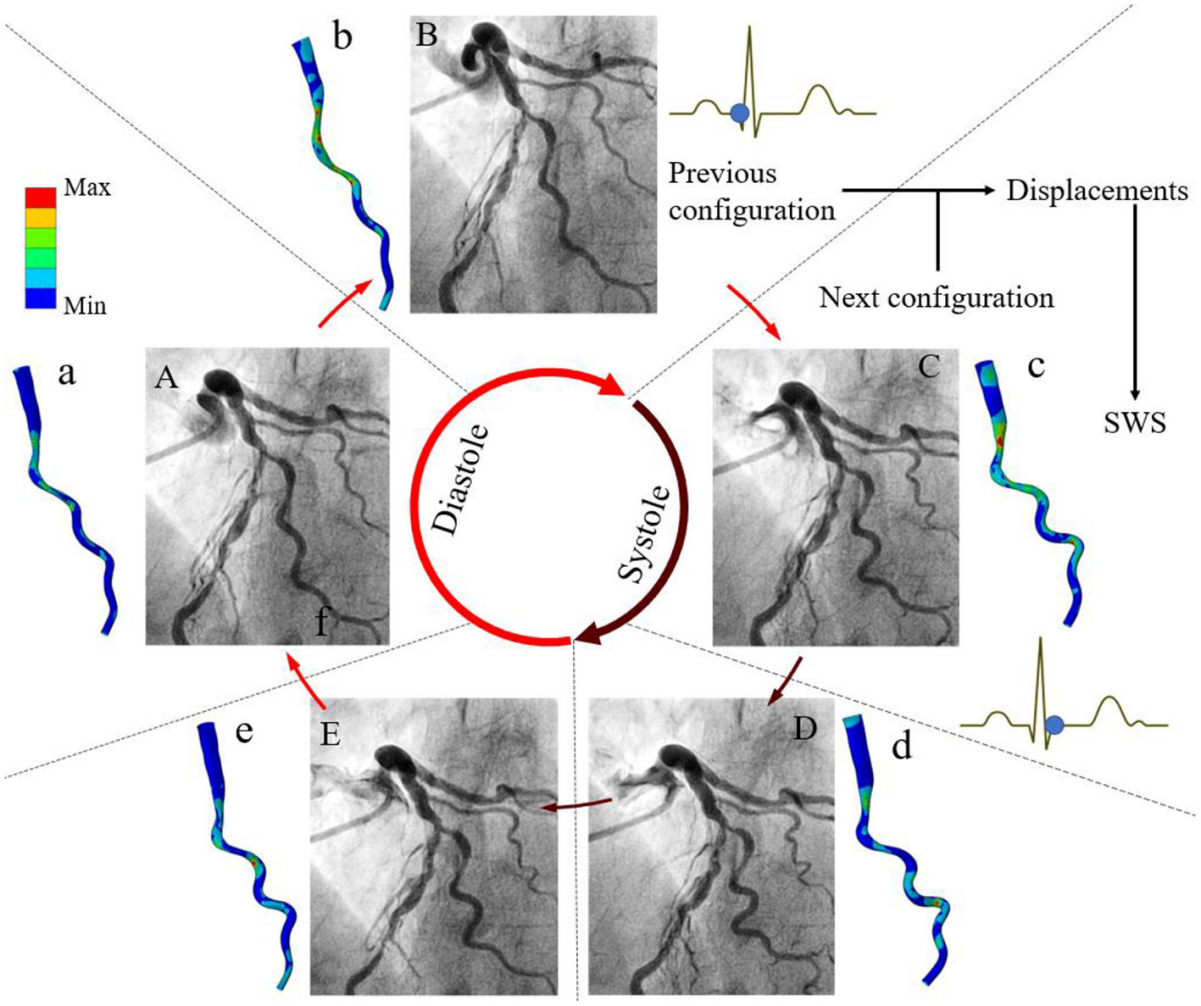
Methods of angiography-based 4D coronary arterial wall dynamics assessment and calculation. Five frames corresponding to specific time-points during the cardiac cycle are identified from the electrocardiogram: **(A)** mid-diastole (diastasis), **(B)** end-diastole, **(C)** early-systole, **(D)** end-systole, and **(E)** early-diastole. Left coronary arteries present large deformations *in vivo*. The local displacements of the superficial wall are determined between these arterial geometries at two consecutive time instants. By using global point-wise displacement mapping relationship on the arteries at the current time-point, the wall strain is determined. Considering the material properties of normal and fibrous tissues, superficial wall stress is further calculated **(a–e)**. Modified from Wu et al. (10).

The concept and application of this method are illustrated in Figure 2. Coronary angiograms with minimal vessel image overlap and foreshortening are selected (13). Several key time-points during the cardiac cycle are identified from the electrocardiogram or according to the different stages of vessel motion during heart contraction and relaxation (Figure 2) (14). The number of frames for cardiac cycle can be determined from one QRS wave to the next on the electrocardiogram, when available. Alternatively, the frame showing the initial clearance of contrast medium from the aorta by contrast-free blood during ventricular ejection identifies early systole. The combination of these signals is used to achieve time synchronization between frames from two single plane angiograms. The vessel geometries are reconstructed and then discretized into structured meshes with identical node dimensions. The point-wise displacements of the deformed arterial wall are determined based on the principle of minimum potential energy. A global displacement function between two consecutive time instants will have its minimum value when all nodes between two consecutive configurations are matched to generate the one-to-one mapping relationship. Because at the stage of diastasis (i.e., mid-diastole) the ventricle is quiescent, the initial configuration for cyclic computation is selected at diastasis, when the kinetic and strain energy of the coronary arteries are at the lowest level throughout the cardiac cycle. Starting from diastasis, the displacements of the vessel wall at the next specific time-point are determined by the mapping relationship. Similarly, the displacements of the vessel wall at subsequent time-points are determined until the next diastasis. The superficial wall strain can be calculated by dividing the element length of structured mesh at the next time-point by that at the previous time-point. When the strain is determined for multiple time-points within cardiac cycle, the stress of arterial wall can be derived based on the assumption of material propeties. The artery was segmented into the normal and diseased parts based on local percent diameter stenosis and assumed to be nearly incompressible, homogenous, isotropic, and hyperelastic as described by Mooney-Rivlin strain energy density function: $W=C_{1}\left(\bar{I_{1}}-3\right)+C_{2}\left(\bar{I_{2}}-3\right)$. Here, *C*_1_ and *C*_2_ are empirically determined material constants. The corresponding material parameters of normal and diseased arterial segments were adapted from a previous study (15). $\bar{I_{1}}$ and $\bar{I_{2}}$ are the first and the second invariant of the unimodular component of left Cauchy-Green deformation tensor.

**Figure 3:**
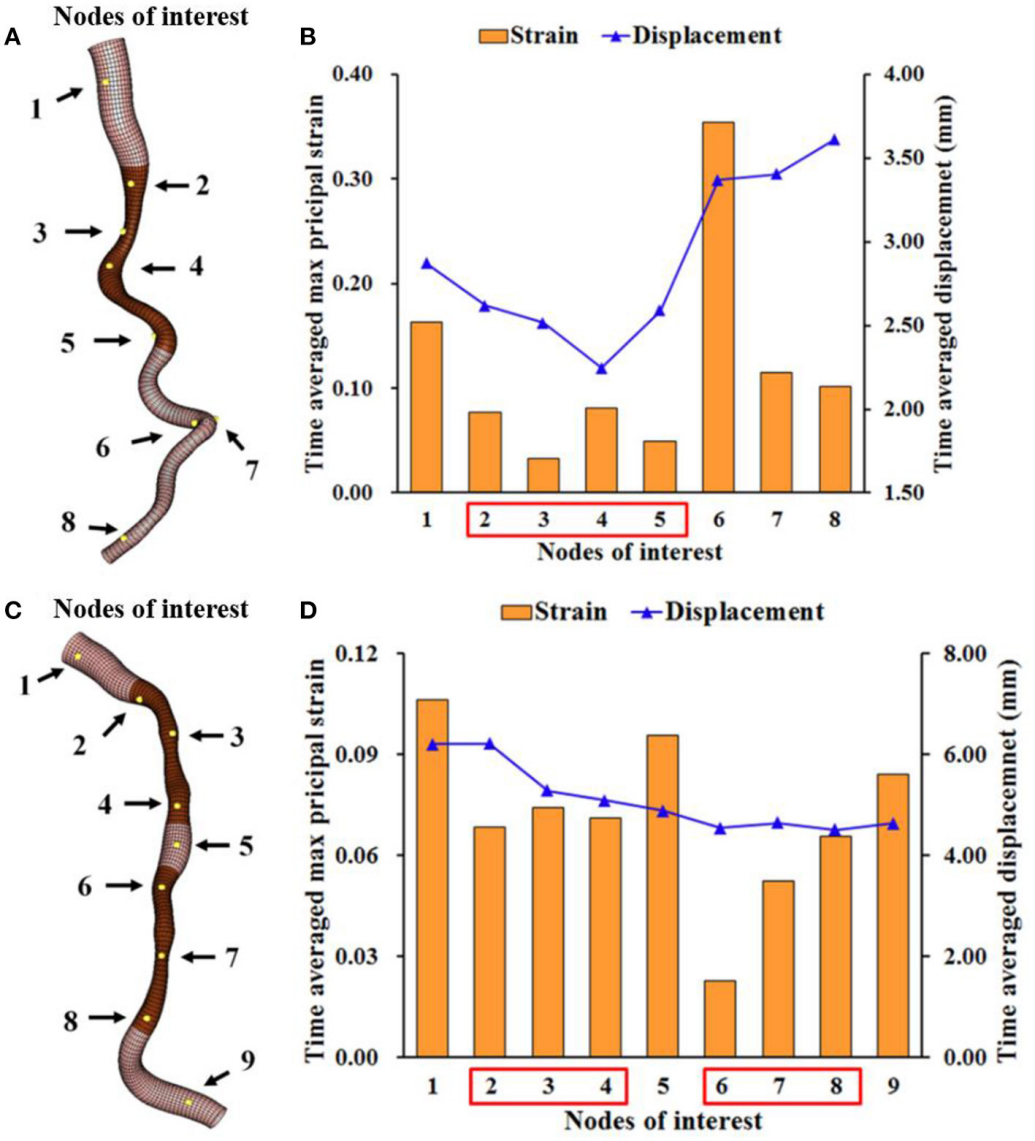
Time-averaged maximum principal strain and displacement at several locations on the LAD and diagonal arteries. The time-averaged maximal principal strain and displacement at the numbered nodes of interest on the diagonal **(A,B)** and LAD **(C,D)** with stenostic segments (dark brown). These nodes at the stenotic segments have lower strain than those at the normal segments.

The clinical feasibility of this approach was first confirmed in a selected case with large coronary artery deformation and motion during cardiac cycle (Figure 2). The calculated motion of the left anterior descending (LAD) and the diagonal artery (Supplementary Videos 1, 2) were consistent with the angiogram (Supplementary Video 3). The LAD moved longitudinally, while the tortuous diagonal artery exhibited remarkable curl motion (10). The time-averaged displacement of LAD (>4.00 mm) was larger than the motion of the diagonal artery (Figure 3). For both coronary arteries, the time-averaged maximum principal strain in the stenotic segment was 5.8%, which was significantly lower (*p* < 0.001) than in the normal segment (12.1%). Although, the locations of the peak wall stress changed with time during cardiac cycle, it was mainly concentrated at the proximal and distal shoulders of the stenotic segments or on the inner and outer walls in segments with large curvature (Supplementary Videos 1, 2) (10). The time-averaged peak wall stress of the LAD was significantly higher than that of the diagonal artery (69.1 ± 11.4 kPa vs. 47.6 ± 8.5 kPa, *p* < 0.001).

## *In Silico* and *In Vivo* Validation Studies

**Figure 4:**
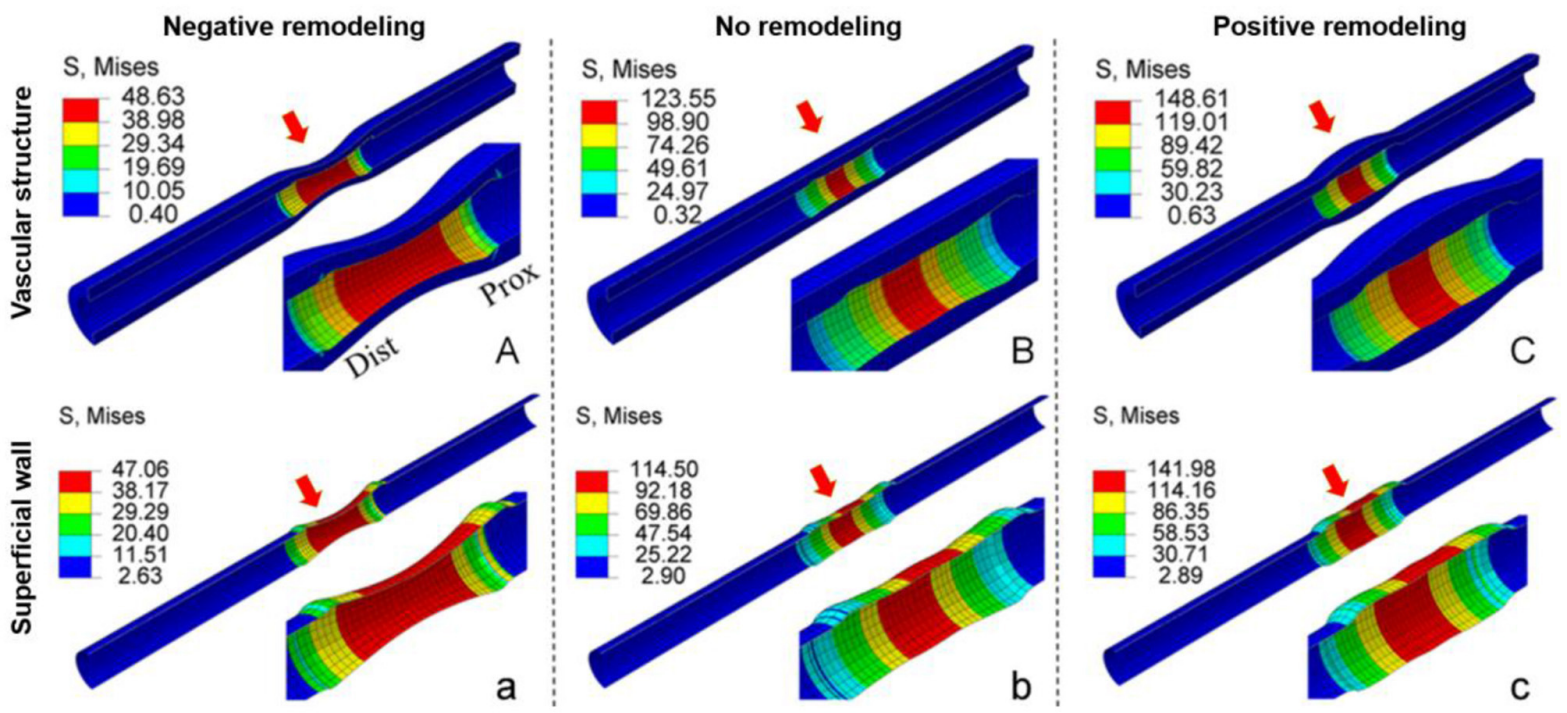
Comparison of the stress distribution of lipid-rich plaque models calculated by the conventional structural mechanical force-based method and superficial wall displacement-based method. There is similar wall stress distribution along the longitudinal superficial wall of the lipid-rich plaque models calculated by the conventional structural mechanical force-based method **(A–C)** and by superficial wall displacement-based method **(a–c)**, regardless of the presence of arterial remodeling. Prox: Proximal; Dist: distal. Six red arrows show the lesion segments of two structural models with three types of arterial remodeling. Modified from Wu et al. (14).

The calculation of wall stress was first validated on *in silico* stenosis models (*n* = 32) (14). The idealized virtual stenosis models were designed with lumen dimensions identical to human coronary arteries (normal lumen diameter of 3 mm). Vessels were 50 mm-long and showed a 10 mm-long concentric stenotic part with a 50% diameter stenosis in its mid-portion. To represent different scenarios of coronary arterial lesions, three different plaque components (i.e., calcified, fibrous, and lipid-rich) and three levels of plaque burden (i.e., 50.9, 62.6, and 69.1 percent volume) were constructed with three types of arterial remodeling (i.e., negative, none, and positive). Our 4D-SWS approach derived from vessel motion *in vivo* was compared with the results of conventional finite element analysis (FEA) with the mechanical force-based method, which generally uses forces as the known conditions to calculate deformation. The maximum wall stress of all types of plaques was not significantly different between both methods (41.6 ± 18.5 kPa vs. 38.9 ± 17.6 kPa, *p* = 0.49). Moreover, there is similar superficial wall stress distribution along the longitudinal superficial wall of all plaque types, including lipid-rich plaque models with three types of arterial remodeling calculated by both methods (Figure 4). The virtual stenotic models with lipid-rich plaques had numerically higher superficial wall stress than calcified and fibrous plaque models, and the superficial wall stress values increased with plaque burden. These results suggest that lipid-rich plaques with positive remodeling (i.e., an adaptive vessel enlargement in response to plaque accumulation) could lead to greater risk of plaque rupture. Indeed, the culprit plaque in patients with acute coronary syndromes (ACS) commonly shows lipid-rich content, high plaque burden, and positive remodeling (16–18).

For *in vivo* validation, our SWS computation procedures were performed on angiographic images from 16 patients with intermediate coronary stenoses included in the Functional Assessment by Various flOw Reconstructions (FAVOR) pilot study (19). Only lesions involving a single vessel segment without image overlap were included for analysis. Coronary segments were defined as normal or stenotic segments based on percent diameter stenosis >20%. The accuracy of superficial wall deformation was validated by comparing the similarity between the centerlines predicted by our 4D approach and the actual centerline that was reconstructed from angiograms at a randomly selected time instant. The morphology of centerline curve derived by these two methods was a good agreement assessed by the classical statistical shape method of Procrustes analysis (scaling: 0.995 ± 0.018 and dissimilarity: 0.007 ± 0.014) (20). This shows that continuous changes in vessel morphology were adequately captured from the analysis of randomly selected cardiac frames (*p* < 0.001). Additionally, in the majority of patients (9 out of 16, 56%) the maximum wall stress was located in the proximal lesion shoulder, and less often in the mid-portion (25%, *n* = 4) or distal shoulder (19%, *n* = 3). This finding is consistent with a previous report showing that plaque rupture occurred most often at the proximal plaque shoulder (21).

## Angiography-Based 4D Coronary Arterial Wall Dynamics: Future Directions and Limitations

Table 1 summarizes the value and limitations of this method and its potential clinical usefulness, which is reviewed in greater detail in the following sections.

**Table 1:** Value and limitations of angiography-based 4D coronary artery dynamic method and clinical usefulness.

**Value and limitations**	
Value	1. Angiography-based solution and potential online availability in the catheterization laboratory.
	2. Realistic reflection of the cyclic motion of arterial wall *in vivo*.
	3. Assessment of the global and local features of the arterial wall with the amplitude and rate of changes in multiple parameters.
Limitations	1. Sensitive to the accuracy of lumen segmentation, especially at location of severe stenosis.
	2. Heart rate-dependent coronary motion.
	3. Further validation of clinical predictive potential needed.
**Potential clinical applications**	
	1. Assessment of the native vessel dynamics
	a. Identification of weak spots in a diseased vessel along the longitudinal direction.
	b. Differentiation of high-risk vessel segments in patients with non-obstructive coronary artery or multivessel disease.
	c. Biomechanical assessment of arterial remodeling, aneurysm formation, and lumen patency.
	2. Assessment of the implanted device dynamics
	a. Assessment of the fracture risk and fatigue life of coronary stents.
	b. Evaluation of the early discontinuity of bioresorbable scaffolds.
	c. Assessment of the effects of wall strain on the patency of (bioresorbable) bypass grafts

### Localization of Coronary Plaque at Risk of Rupture and Prediction of Future Events in Patients With Mild or Non-obstructive Coronary Artery Disease (NOCA)

Histopathological post-mortem studies in victims of sudden coronary death demonstrated that acute thrombi are associated in 55–65% with the rupture of a thin fibrous cap atheroma (22). In patients undergoing primary PCI after STEMI, there is a significant residual risk of adverse events caused by non-target and non-flow limiting lesions (called NOCA) that do not need stenting at the time of the initial procedure (23). For example, the 2-year results of RESOLUTE All Comers trial demonstrated that approximately half of the patient-oriented composite endpoint (all-cause death, myocardial infarction, revascularization) might be attributed to the progression of lesions in the non-target vessels or in the target vessels outside of the culprit segments at index event (24). If these high-risk lesions were identifiable during the initial procedure, targeted pharmacological, or mechanical interventions could be applied to mitigate the risk of future ACS and to prevent recurrent heart attacks in these patients.

**Figure 5:**
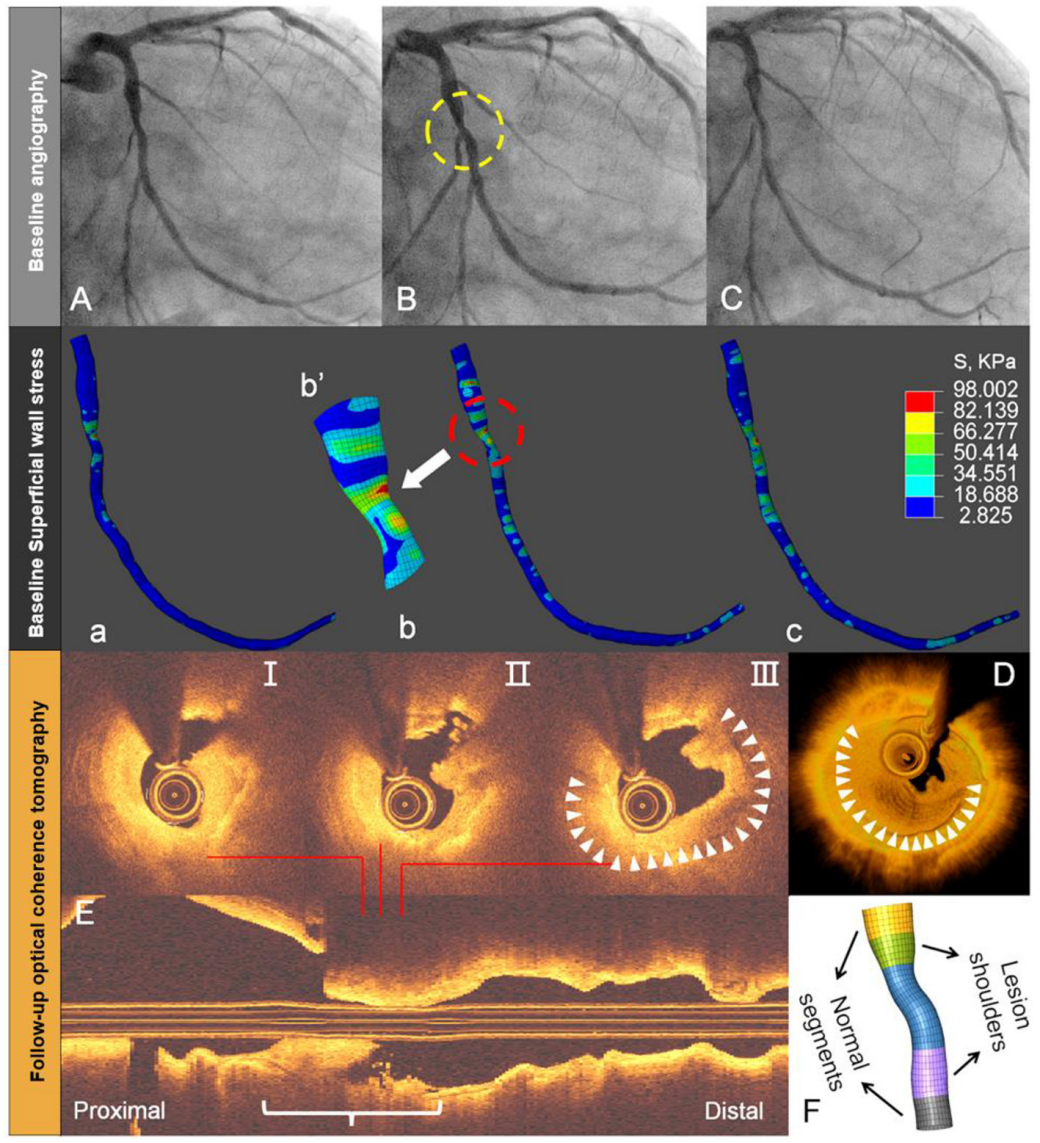
Angiography-based superficial wall stress on diagnostic angiography and late plaque rupture. Baseline angiography shows (yellow circle) an intermediate mid-LCx lesion **(A–C)**. Superficial wall stress, calculated by the 4D approach at baseline angiography, reveals more local stress concentration in the stenotic segment (white arrow) or throat site **(a–c)**. This location corresponds with the site of lumen irregularity, thrombus, and plaque rupture during late acute coronary syndrome, as shown by OCT on selected cross-sections **(I-III)**, 3D rendering **(D)**, and longitudinal OCT **(E)**. The reconstructed throat segment and lesion shoulders are shown along with percent diameter stenosis **(F)**. Modified from Wu et al. (11).

Although, several attempts have been made to establish the criteria that define such rupture-prone plaques using cardiac imaging, the absolute event rates predicted by intravascular imaging remain low under the current best of medical treatment (25, 26). In addition, systematic imaging of all three coronary arteries failed to be clinically cost-effective (27). Theoretically, plaque rupture represents the structural failure of its fibrous cap due to excessive mechanical stress or strain. Therefore, several studies of coronary image-based computational modeling attempted to improve the predictive accuracy by incorporation of mechanical indices (28–30). The threshold value of 300 kPa is commonly used as a high-stress value from the experimental fracture *in vitro* of human plaque caps (1). However, plaque rupture might be triggered at a lower level than this threshold, because coronary artery *in vivo* is a typical fatigue environment with high-cycle and low-level stress due to the repetitive cardiac contraction (31). Note that the stress and strain values of human coronary artery *in vivo* are significantly lower than those of *in vitro* failure. Under physiological conditions, the axial strain and stress of coronary arteries are up to 62% and 200 kPa (32). We recently reported two cases where the maximum superficial wall stress computed from a diagnostic angiogram using our method was co-localized with the sites of late plaque rupture, as confirmed by optical coherence tomography (OCT) during ACS several months later (Figure 5, case 2) (11).

**Figure 6:**
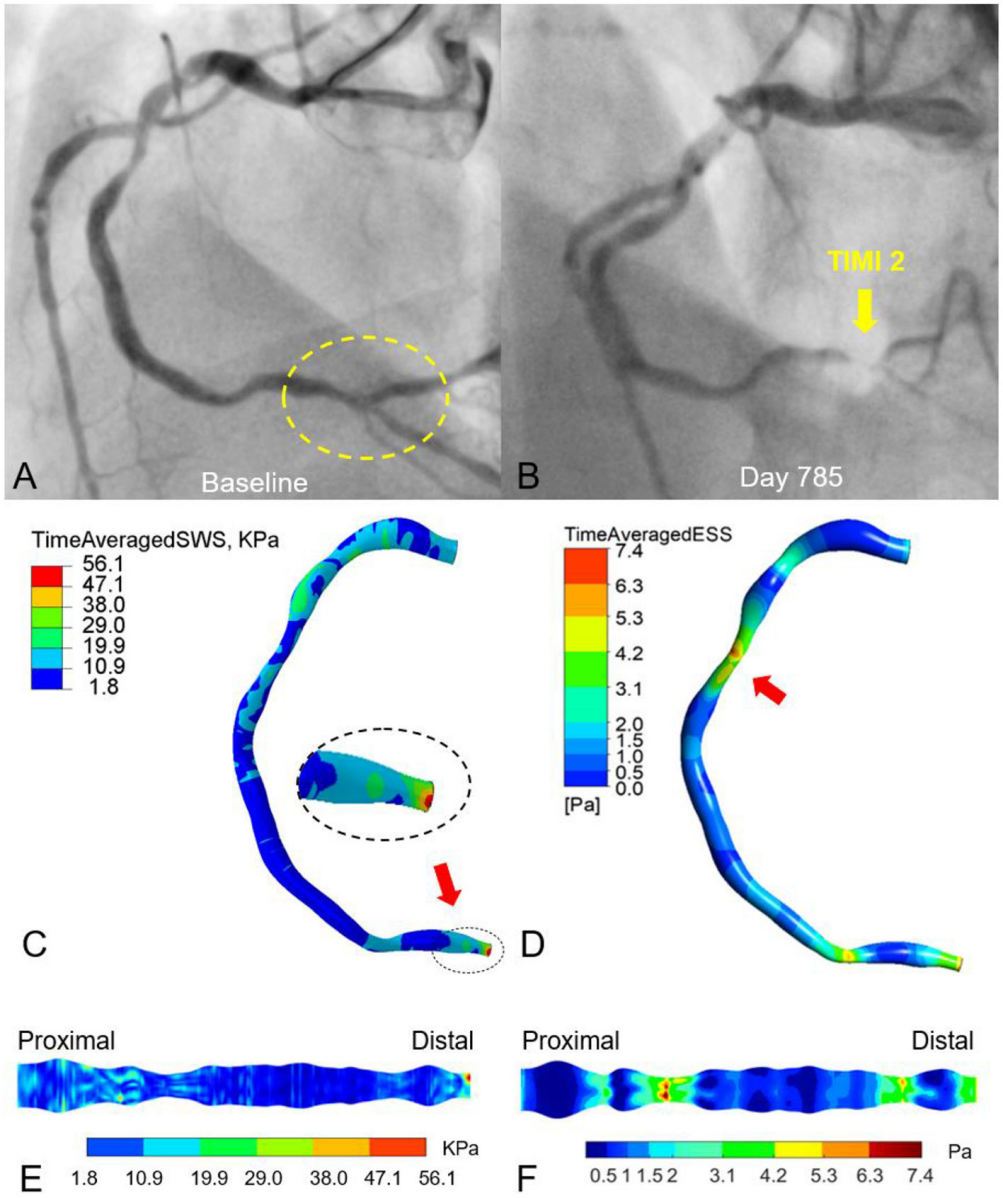
Effects of vessel deformation-induced superficial wall stress and fluid-induced endothelial shear stress on plaque progression and clinical adverse events. The moderate lesions in proximal and distal RCA remained untreated according to physiological guidance with iFR **(A)**. On day 785, the patient was admitted to the hospital due to recurrent angina. Angiography demonstrated the progression of the distal RCA stenosis with impaired TIMI-2 coronary flow, suggesting plaque progression as well as potential atheroma rupture **(B)**. The time-averaged superficial wall stress was relatively high at the distal RCA with 56 kPa **(C)** and co-located with the site of late plaque rupture **(B)**. **(D)** Relatively high time-averaged endothelial shear stress (ESS) (6–7 Pa) was located at the stenotic segments of proximal and distal RCA (lesions left untreated), while the mid-segment exhibits a very low ESS (<1 Pa). **(E,F)** Expanded views of superficial wall stress and ESS distribution.

Figure 6 shows another example to suggest the association between superficial wall stress and newly developed stenosis at follow-up. After physiological assessment during index PCI (Figure 6A, baseline angiography), RCA showed preserved iFR value and the stent implantation in the proximal and distal lesions was deferred. On day 785, the patient was admitted for recurrent unstable angina. Repeat angiography (Figure 6B) showed that RCA had only a TIMI grade 2 flow, which was predominantly caused by the progression of the distal lesion. The initial diagnostic coronary angiogram was analyzed by both superficial wall stress (Figure 6C) and ESS (Figure 6D) to investigate their impacts on possible plaque progression. In contrast to superficial wall stress calculated based on the deformation of coronary artery during cardiac cycle, ESS is the friction between intravascular blood flow and endothelial layer, and was analyzed by computational fluid dynamics. In Figure 6C, the high level of time-averaged superficial wall stress was found at the site of distal stenosis with rapid progression (Figure 6B, yellow arrow). Figure 6D shows that the high time-averaged ESS is located at the stenotic segments RCA (lesions left untreated), while the mid-segment exhibits a very low ESS (<1 Pa). The expanded view shows that ESS is correlated with lumen diameter (Figure 6F). These observations illustrate the potential of angiography-based superficial wall stress for the identification of rupture-prone plaque. With further prospective validation, this technique can hopefully inform personalized patient care with optimized pharmacological therapy or local device-based plaque modification.

### Assessment of the Mechanical Failure Risk of Coronary Stents

Histopathological studies have demonstrated that stent fracture is one of the potential causes of drug-eluting stent failure (33). Such mechanical failure, which typically occurs at kinking points in long or overlapping stents (34), is often the consequence of dynamic forces that result from the motion of coronary arteries. Once implanted, coronary stents will be exposed to cyclic deformation at least 86,400 times per day. Therefore, the indices of dynamic arterial morphology may be useful to estimate stent failure risk (35–37). For example, Girasis et al. (36, 37) analyzed the dynamic changes in 3D bifurcation angle (BA) of the left main coronary artery. After bifurcation stenting with a two-stent strategy, the proximal BA became larger and the distal BA narrower (36). A systolic-diastolic distal BA range < 10° (i.e., a more rigid, “full metal jacket” bifurcation) had significantly higher adverse event rates (50.8 vs. 22.7%, *p* < 0.001) and was associated with a higher 5-year adverse event rate (hazard ratio: 2.65, *p* < 0.001) (37).

**Figure 7:**
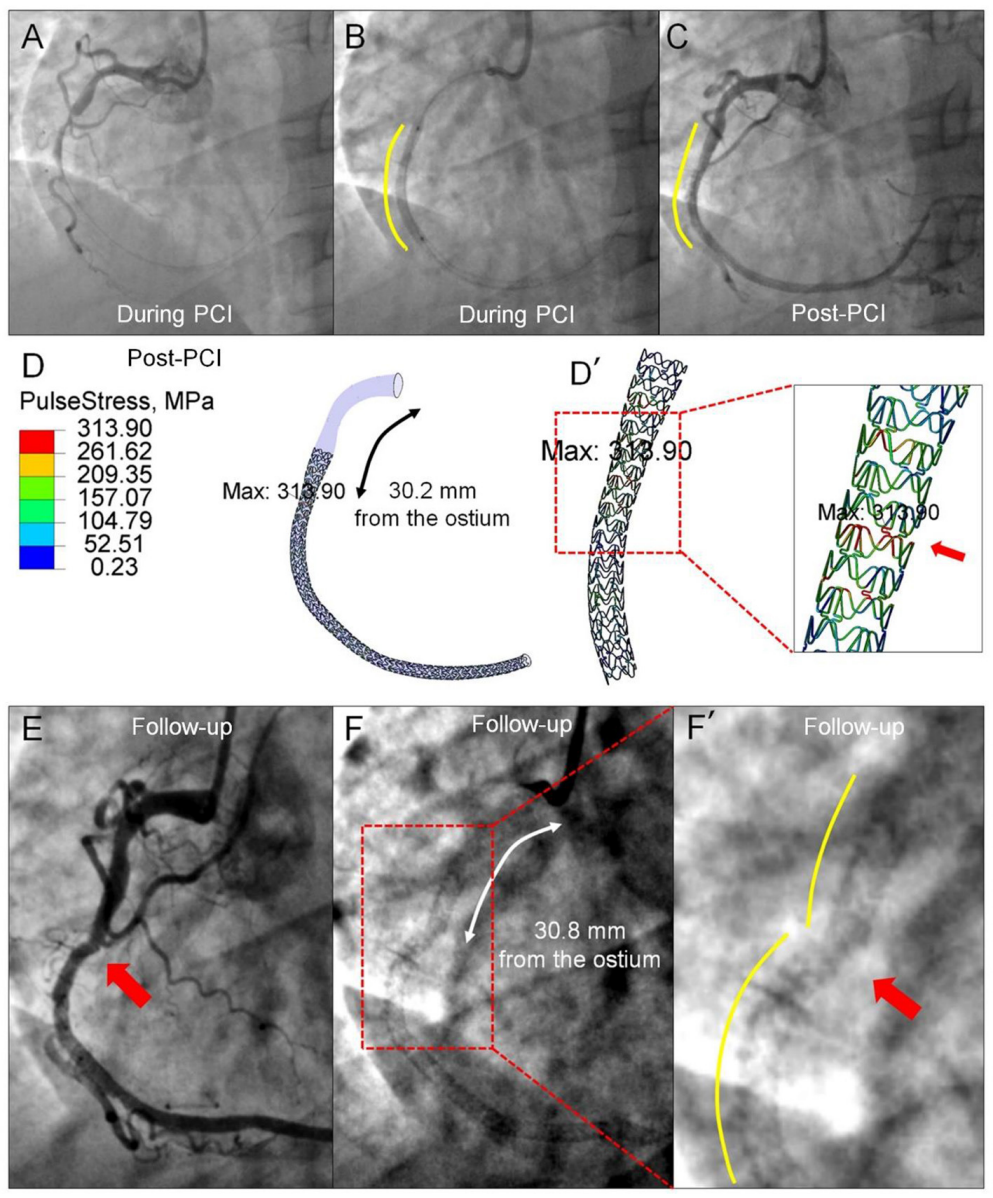
Prediction of late stent fracture by the pulse stress on the implanted stent using angiography-based 4D coronary artery dynamic method. **(A)** Angiography prior to implantation shows a total occlusion of the mid-segment of the RCA and a smooth curve of the implanted stent. **(B)** Visible contours of the inflated balloon delineate the proximal and distal ends of the implanted stent (Xience V). **(C)** Straightening of the proximal segment occurs following stent implantation. **(D)** The highest pulse stress (313.90 MPa), derived from the difference between the stress state at end-systole and end-diastole, is found at the site of 30.2 mm from the ostium. [**(D')**, red arrow] The zoomed view shows the pulse stress distribution of the implanted stent. Angiograms 20 months later show stent fracture **(E)** located at 30.8 mm from the ostium, resulting in luminal irregularity **(F)**. The site of the highest pulse stress calculated by the angiography-based 4D dynamic method co-localizes with the stent fracture site [**(F')**, red arrow] documented 20 months later.

Although, it is recognized that repetitive and fluctuating stress, induced by the dynamic deformation of coronary arteries, is an important mechanism of stent fracture, quantitative analysis on *in vivo* mechanical stress in association with a stent fracture using FEA is still limited (38). By using the angiography-based 4D dynamic method, it becomes relatively easy to calculate the dynamic stress on implanted stents *in vivo*. In Figure 7, the highest pulse stress on the stent, derived from the difference between the two stress states at end-systole and end-diastole, is localized 30.2 mm from the coronary ostium at the index procedure, at the exact location where stent fracture occurred 20 months later (30.8 mm from the ostium). This approach might be useful to estimate the stress distribution of devices and to predict the fracture risk when implanted in the specific coronary arteries with different deformation.

### Relationship Between Dynamic Parameters, Vascular Remodeling, and Lumen Patency

Vascular remodeling can be considered as a dynamic functional and morphometric adaptive process of a vessel in response to biomechanical stimuli that lead to changes in vascular structure and properties (39). A vessel composed of smooth muscle cells, elastin, and collagen fibers, each with different elastic performances, has to maintain its dynamic stability over a wide range of diameter variations under normal and abnormal biomechanical conditions. The chronic expansion and shrinkage of the vessel lumen are theoretically regulated by biomechanical stresses that result from the equilibrium relationship between transmural pressure *P* and circumferential stress σ_θ_, which can be computed by the Laplace's law σ_θ_ = Pr/*t*, where, *r* is lumen radius and *t* is wall thickness. Generally, lumen narrowing appears when plaque burden exceeds about 40% of the vessel “normal” cross-sectional area, as defined by the external elastic membrane (40). Then, adaptive remodeling becomes exhausted, plausibly because the gradually increased circumferential stress counterbalances the transmural pressure, resulting in plaque encroachment on the lumen. In contrast, if transmural blood pressure dominates the process of vascular remodeling, vessels can undergo outward expansion and become even larger. In particular, vein grafts, transplanted from their native low-pressure environment, cannot bear the elevated circumferential stress caused by the pressure in the systemic arterial circulation (from ~10 to ~100 mmHg).

It has been suggested that cyclic motion of coronary bypass grafts plays an important role in lumen patency (41). The compliance mismatch at the anastomosis site between the distal end of the graft and the native artery could increase local wall strain and stress, leading to cumulative regional graft injury. A previous experimental study revealed that constraining graft wall motion by the use of an external casting stent may reduce the progression of graft disease (42), although, this kind of static casting might also impede flow through vasa vasorum and cause graft hypoxia. In the long-term, both mechanisms could result in post-necrotic neointimal overgrowth. Therefore, bioresorbable bypass grafts armored with nitinol rings in their wall could serve as a template to the restorative process of “Mother Nature.” Currently, such innovative technology is being tested in an animal model and initial First-in-Human studies (NCT04545112) (43). The angiography-based examination of graft dynamics in this pre-clinical model, allows to detect weak spots or regions of high strain. These weak spots could be amended by local reinforcement of the graft wall, by changing the curvature of the graft, or by modifying the angulation of the anastomosis. Exploring the relationship between dynamic mechanical parameters and vascular remodeling and lumen patency may equally allow to quantify the process of vascular remodeling in native coronary arteries.

Many approaches have been evaluated for the prediction of events and the estimation of plaque propensity for rupture, thrombosis, or progression, including intracoronary imaging and image-based computational modeling. Table 2 summarizes the strengths and limitations of relevant imaging modalities and imaging-based computational modeling techniques and application scenarios. From a biomechanical viewpoint, ESS (28), a friction stress caused by flowing blood acting on the wall, is calculated on a static model of coronary arteries at end-diastole from 3D reconstruction of angiography or in combination with intravascular images, such as ANGUS (44). The SWS approach instead focuses on the dynamic mechanical status of the superficial wall layer and is directly calculated from the deformation of arteries based on angiography during the entire cardiac cycle. Plaque structural stress (2, 30), which is calculated from plaque composition and architecture on intravascular cross-sectional images, estimates plaque stress status under the prevailing coronary arterial pressure. Previously reported techniques, such as elastography (45) and palpography (46), measure plaque strain *in vivo* based on the deformation of IVUS images under blood pressure. Since major adverse cardiovascular events (MACE) represents a combination of different clinical endpoints resulting from several different mechanisms, comparative evaluation of imaging-based computational modeling for MACE prediction requires further exploration. Further clinical validation of the angiography-based SWS is needed in order to demonstrate its incremental value for the prediction of future events, compared with other imaging modalities and conventional risk factors (e.g., age, gender, diabetes, etc.). Larger studies investigating the correlation between the distribution of SWS and clinical events are needed.

**Table 2:** Summary of cardiac imaging and computational modeling techniques.

**Categories**	**Techniques**	**Theoretical strengths**	**Limitations**	**Application scenarios**
Cardiac imaging	Angiography	• High spatial resolution (150~250 μm) • High temporal resolution (33~80 ms) • Dynamic blood flow • Dynamic motion and deformation of coronary tree	• Lacking 3D information	• Diagnosis of coronary artery anomalies and guide interventional therapy • 3D reconstruction of artery and centerline • 4D reconstruction of arterial dynamics
	IVUS	• High penetration depths for assessing plaque burden and detecting lumen size	• Low spatial resolution (axial: 100~150 μm; lateral: 150~300 μm) • Limited for assessing strut malapposition and detecting thrombus	• Measurement of lumen and vessel dimensions, lesion characterizations • Guide interventional therapy
	OCT	• High spatial resolution (axial: 10~20 μm; lateral: 20~90 μm) for accurately detecting lumen, thrombus, or stent-related morphologies	• Low tissue penetration depths (~2 mm) • Limited for assessing plaque burden and detecting vessel size	• Measurement of lumen dimensions, lesion characterizations, evaluation of strut-level • Guide interventional therapy
	NIRS	• Quantitative assessment of lipid core burden index	• Limited for plaque structure and cap thickness	• Detection of lipid-rich plaque
Image reconstruction	ANGUS	• More accurate for 3D reconstruction model of vessels	• Need angiography and IVUS • 3D reconstruction only at end-diastole	• For endothelial shear stress analysis
Image-based computational modeling	ESS	• Assessing the hemodynamics of near-wall with a profound influence on vascular biology based on angiography or combined with intravascular images	• Static assessment at end-diastole	• Assessing plaque progression and thrombogenesis
	SWS	• Measuring the dynamics of the superficial wall base on angiography • Dynamic mechanical behavior of coronary arteries during a cardiac cycle	• Sensitive to arterial geometry • Heart rate-dependent coronary motion	• Assessment of the native vessel dynamics • Assessment of the implanted device dynamics (Detail see Table 1)
	PSS	• Assessing the stress state of the plaque structure based on IVUS	• Segmentation of detail plaque components • Require blood pressure and mechanical properties of plaque components	• Assessment of plaque rupture risk
	Elastography/palpography	• Measuring plaque strain *in vivo* based on IVUS	• Sensitive to heart beating and the location of imaging sensor	• Detection of the vulnerable plaques with a high strain region at the surface in the close vicinity of low strain regions

*ESS, endothelial shear stress; IVUS, intravascular ultrasound; NIRS, near-infrared spectroscopy; OCT, optical coherence tomography; PSS, plaque structural stress; SWS: superficial wall strain/stress*.

## Conclusions

*In silico* and *in vivo* studies have revealed that angiography-based assessment of dynamic coronary artery deformation allows computation of superficial wall strain and stress, as reported by Wu et al. (10, 11, 14). Lipid-rich plaque models had numerically higher wall stress than calcified and fibrous plaques, and wall stress increased with plaque burden. In selected observations, high wall stress spots at baseline co-localize with the site of plaque rupture in patients with late clinical events. By the identification of weak spots over the full length of the diseased coronary artery tree, it may become possible to assess the fracture risk and fatigue life of implanted stents, and to explore biomechanical mechanism of arterial remodeling and lumen patency. Because this technique only requires angiographic data, essential steps (including high-precision and automatic lumen segmentation, vessel reconstruction, anatomical landmarks and cardiac frames detection) can be implemented in a seamlessly integrated workflow. As a result, the superficial wall strain and stress analysis can potentially become an available online tool in the catheterization laboratory in the future.

## Author Contributions

XW, WW, PS, and YO conceived the idea and wrote the first draft. All authors contributed substantially to the discussion of content and reviewed/edited the manuscript before submission.

## Conflict of Interest

CB reports institutional research grants (Thoraxcentrum Twente) from Abbott Vascular, Biotronik, Boston Scientific, and Medtronic, outside the submitted work. ST reports institutional research grants from Pulse medical imaging technology, Shanghai, China. WW reports institutional research grant and honoraria from MicroPort. The remaining authors declare that the research was conducted in the absence of any commercial or financial relationships that could be construed as a potential conflict of interest.

## Supplementary Material

Supplementary Video 1 Superficial wall dynamics of the left anterior descending. The LAD moved longitudinally, and the peak superficial wall stress was mainly located at the proximal and distal shoulders of the stenotic segments during the cardiac cycle.

Supplementary Video 2 Superficial wall dynamics of the diagonal artery. The tortuous diagonal artery exhibited remarkable curl motion, and the peak superficial wall stress was located at the proximal and distal shoulders of the stenotic segments or on the inner and outer walls in segments with large curvature during the cardiac cycle.

Supplementary Video 3 Coronary angiogram of a selected case with large deformation and motion. The angiogram shows the LAD with diffuse lesion and the diagonal artery with narrowing at ostium.

## Abbreviations

ACS: acute coronary syndrome
BA: bifurcation angle
ESS: endothelial shear stress
FEA: finite element analysis
IVUS: intravascular ultrasound
LAD: left anterior descending
MACE: major adverse cardiovascular events
NIRS: near-infrared spectroscopy
OCT: optical coherence tomography
PCI: percutaneous coronary intervention
PSS: plaque structural stress
RCA: right coronary artery
SWS: superficial wall strain/stress
4D: four-dimensional.
